# Physical activity and depression of Chinese college students: chain mediating role of rumination and anxiety

**DOI:** 10.3389/fpsyg.2023.1190836

**Published:** 2023-07-31

**Authors:** Yanying Liu, Qingkun Feng, Kelei Guo

**Affiliations:** School of Physical Education and Health, Zhaoqing University, Zhaoqing, China

**Keywords:** physical activity, depression, rumination, anxiety, college students

## Abstract

**Objective:**

To explore the relationship between physical activity and depression among college students, as well as the mediating role of rumination and anxiety.

**Methods:**

A total of 1,292 Chinese college students were investigated by physical activity questionnaire, rumination scale, self-rating anxiety scale (SAS), and depression scale.

**Results:**

(1) There was a significant negative correlation between physical activity and depression (r = −0.399, *p* < 0.01), and the direct path of physical activity on depression was significant (*β* = −0.399, *t* = −13.374, *p* < 0.01). (2) Physical activity negatively predicted rumination (*β* = −0.322, *t* = −10.440, *p* < 0.01) and anxiety (*β* = −0.222, *t* = −7.089, *p* < 0.01). Rumination positively predicted anxiety (*β* = 0.283, *t* = 9.017, *p* < 0.01) and depression (*β* = 0.267, *t* = 9.046, *p* < 0.01). Anxiety positively predicted depression (*β* = 0.262, *t* = 8.902, *p* < 0.01). (3) Rumination and anxiety play a significant mediating role between physical activity and depression. The mediating effect involves three paths: physical activity → rumination → depression (the mediating effect value: −0.076); physical activity → anxiety → depression (the mediating effect value: −0.052). Physical activity → rumination → anxiety → depression (the mediating effect value: −0.021).

**Conclusion:**

(1) Physical activity can negatively predict the rumination, anxiety, and depression of college students, which means physical activity can reduce rumination, anxiety, and depression of college students. (2) Physical activity can not only directly affect the depression of college students, but also indirectly affect depression through the independent intermediary role of rumination and anxiety, and the chain mediation of rumination and anxiety.

## Introduction

Depression refers to a sub-syndrome involving depressive and negative emotional symptoms (Almeida et al., 2022; Vidal-Arenas et al., 2022). The main clinical manifestations are low mood, emotional excitement, loss of interest, anhedonia, physical sickness, sleep disorder, inappetence, slow thinking, low self-esteem and suicidal tendency, severely interfering the work and life of patients and their families, and imposing substantial burdens on society. According to the World Health Organization, depression has become a common mental illness worldwide, with approximately 280 million people suffering from varying degrees of clinical depression, accounting for about 4% of the global population [World Health Organization (WHO), 2022]. Depression is predicted to be the leading cause of disability and death by 2030 across the world (Lépine and Briley, 2011). College students are in early adulthood (aged between 18 and 29 years), an important developmental stage characterized by significant social and psychological changes (Nelson and Chen, 2007; Kuang et al., 2022). Since the early adulthood is a critical developmental period with unique features, including higher-order cognitive abilities, identity exploration, self-focus, instability, and possibilities (Nelson and Chen, 2007), college students are susceptible to experiencing depressive disorder, in response to academic stress and life pressure, who have become a high prevalence group of depression. It is found that in recent years, the prevalence of depression among Chinese university students is as high as 20%–60% (Wang et al., 2021). College students with depression have difficulty in communicating with others and sleeping, lose interest in learning and life, which could be accompanied by thoughts of death (Liu et al., 2019). Thus, it is one of the priorities to explore the key influencing factors and related mechanisms of depression among college students, to conduct timely and effective prevention and intervention, so as to improve their mental health.

### Physical activity and depression

Treating and preventing depression remain a public health strategy (Cuijpers et al., 2012). Physical activity refers to any body movement triggered by skeletal muscles requiring energy expenditure (Xu, 2014), affecting a range of biological and psychosocial processes, and being involved in the pathophysiology of depression (Kandola et al., 2019). Exercise, as a subset of physical activity, involved in biological and psychosocial processes, influences the pathophysiology of depression (Kandola et al., 2019). Physical activity contributes to treating and preventing depressive symptoms (Kandola et al., 2019). Chi et al. (2021) found that both highly and moderately active physical activities had significant health benefits for relieving depressive symptoms. Rodriguez-Ayllon et al. (2021) revealed a negative correlation between moderate to vigorous physical activity and depression. Depressed patients tend to be less physically active than non-depressed individuals, while increased aerobic exercise or strength training has been shown to reduce depressive symptoms significantly (Paluska and Schwenk, 2000). Pearce et al. (2022) observed an inverse curvilinear dose–response association between physical activity and depression, with steeper association gradients at lower activity volumes. A narrative review pointed out the bidirectional relationship between physical activity and adolescent mental health (Pascoe and Parker, 2019). Research findings have shown that regular physical activity played a crucial role in preventing mental illnesses among college students, especially among those facing various challenges (Zhang et al., 2022b). Conversely, lack of exercise seems to increase risks for psychological disorders. Sedentary behavior, such as too much sitting, increased stress, anxiety, and depression of university students (Lee and Kim, 2019). As Wu et al. (2015) investigated, there are independent and interactive associations of low physical activity and high screen time with increased risks of mental health problems and poor sleep quality among Chinese college students.

Although the impact of physical activity on depression has been widely reported in recent years, the internal mechanism and mediating effects of physical activity on depression have not been fully revealed. By studying the relationship between physical activity and depression among college students, as well as the mediating role of rumination and anxiety, this paper aims to further explore the effect of physical activity on depression among college students, and reveal the mediating role of physical activity, thus providing theoretical guidance for the prediction and prevention of college students’ depression. Hypothesis 1 is presented as: physical activity has a significant negative predictive effect on depression among college students.

### The mediating role of rumination

Rumination refers to the involuntary and repetitive thoughts on the causes and consequences of negative events or emotions that an individual suffered, which prevent the person from coping with the problems (Nolen-Hoeksema, 1991). Generally, rumination is a repetitive negative mode of thinking. Studies have shown that rumination is moderately or highly associated with depression (Rood et al., 2009; Olatunji et al., 2013). When individuals are immersed in negative emotions, they will repeatedly reflect the causes and fall into thinking traps, leading to depression (Nolen-Hoeksema et al., 2008; McLaughlin and Nolen-Hoeksema, 2011), while such negative emotions will stimulate individuals to form more false cognitive biases (Lyubomirsky et al., 1999). People with high levels of rumination tend to experience major depressive disorder since they focus on the negative experience without thinking about the feasible solutions (Nolen-Hoeksema and Morrow, 1991). As suggested by the self-regulatory executive function model, rumination is a well-established risk factor for the onset of negative emotions such as depression and anxiety (Nordahl et al., 2019). The response styles theory was proposed to define rumination as a passive and repetitive style of responding to negative mood, because patients focus on their depressive symptoms without taking actions to solve the problems they faced (Nolen-Hoeksema et al., 2008). It is found that rumination prolongs and exacerbates negative emotions and depressive symptoms, impairs problem solving, and interfering with cognitive function (Ning et al., 2015), which plays a negative role in the rehabilitation of depressed patients in cognitive behavioral therapy. Hence, rumination tends to have an important predictive effect on depression among college students.

In recent years, scholars and researchers have paid more attention to the influence of physical activity on rumination. Physical activity was proved to be effective as monotherapy for rumination (Cooney et al., 2013). Mental and physical training (MPA), combining meditation and aerobic exercise, is an effective intervention for reducing depressive symptoms and rumination (Demmin et al., 2022). Alderman et al. (2016) examined the efficacy of an 8-week intervention of MAP training in improving symptoms of depression and rumination in patients with major depressive disorder. Brand et al. (2018) found that single bouts of moderately intense exercise exerted a positive impact on the rumination of individuals with mental disorders, and further improvements were observed in those engaging in physical activities for the second time. Schmitter et al. (2020) pointed out that rumination, self-esteem, and memory bias are possible mediators in the treatment of depression through physical activity. Physical activity moderates rumination, and rumination can predict depression. Hypothesis 2 is presented as: rumination plays a mediating role between physical activity and depression among college students.

### The mediating role of anxiety

A link has been observed between anxiety and depression. Despite they have some overlapping symptoms, anxiety and depression are different conditions. Some researchers claimed that many individuals had both anxiety and depression. The National Mental Illness Comorbidity Survey conducted by the Survey Research Center of Michigan University in the United States found that the comorbidity of major depression and anxiety disorders reached 50% (lifetime comorbidity rate) or 51.2% (one-year comorbidity rate), that is, more than half of the patients with major depression have an anxiety disorder in their lifetime or within the same year. About 85% of individuals with depression experience significant anxiety, while 90% of patients with anxiety have serious depression (Tiller, 2013). According to the National Comorbidity Survey (NCS), 51.2% of patients with depression had comorbid anxiety disorders in the past 12 months, and 23.7% of patients suffering from anxiety had symptoms of depression (Kessler et al., 1994). Therefore, anxiety is identified as one of the potential predictors of depression.

Physical activity tends to be protective against anxiety disorders (Paluska and Schwenk, 2000). Studies have shown a significant negative correlation between physical activity and anxiety, which remains valid after controlling the interference factors (Horvath et al., 2019). Evidence suggests that participating in physical activity protects against anxiety disorders, except in case series and small uncontrolled studies (Ströhle, 2009). Epidemiological data shows that more active people are less likely to have anxiety disorders. As pointed out by Stubbs et al. (2017), physical activity is beneficial for the treatment of anxiety disorders. In addition, systematic reviews claimed that exercise training, a subset of physical activity, can alleviate the symptoms of anxiety and stress-related disorders. Physical activity is an effective approach to addressing anxiety symptoms in the young. Murray et al. (2022) found that a web-based aerobic resistance exercise intervention and a web-based yoga mindfulness exercise intervention are effective methods for relieving college students’ depression and anxiety. On the contrary, low physical activity and high screen time increase the risks of mental health problems, such as anxiety, depression, psychopathological symptoms, and poor sleep quality (Wu et al., 2015; Cahuas et al., 2020). Previous studies suggest that increasing physical activity is beneficial for preventing and improving anxiety (Carter et al., 2021). It can be concluded that physical activity has a significant negative predictive effect on the anxiety among university students. Therefore, hypothesis 3 is proposed as: anxiety is the mediating variable between physical activity and depression in college students.

### Chain mediating role of rumination and anxiety

Rumination is a process of uncontrolled and narrowly focused negative response, which motivates individuals to reflect the causes and consequences of negative events and is a hallmark of anxiety. The response styles theory (Nolen-Hoeksema, 1991; Nolen-Hoeksema et al., 2008) was proposed to explain the insidious relationship between rumination and depression. Individuals with higher levels of rumination are more likely to dwell on the causes and potential adverse consequences of problems in times of distress, which reduces individual self-efficacy, increases individual anxiety, and makes individuals immersed in the negative self-perception of “being inferior to others” and the negative emotions of loss and helplessness, thereby increasing the possibility of depression. That is, rumination exacerbates depression, enhances negative thinking, impairs problem-solving, and eventually erodes social support. Anxiety is found to be directly associated with rumination (Arslan et al., 2022). Sun et al. (2022) stated that rumination was independently associated with anxiety and depression. A study on anxiety/depression symptoms in early adolescence revealed that rumination accounted for a certain proportion of the changes in anxiety/depression in later stages (Hansell et al., 2022). Rumination can positively predict anxiety. Thus, hypothesis 4 is proposed that rumination and anxiety play a chain mediating role between physical activity and depression.

### Hypotheses of this study

**Figure 1:**
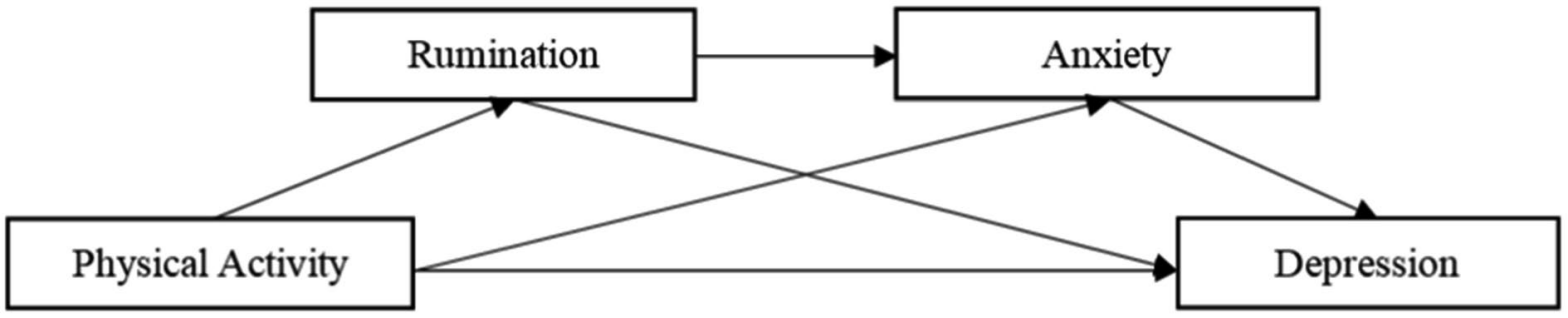
The hypothetical structure model.

To sum up, to investigate the internal mechanism between physical activity and depression, this study aims to build a chain mediating model (as shown in Figure 1) and will verify the following aspects: (1) Physical activity can negatively predict depression of college students; (2) Rumination plays an independent intermediary role between physical activity and depression of college students; (3) Anxiety plays an independent intermediary role between physical activity and depression of college students; (4) Rumination and anxiety play a chain intermediary role between physical activity and depression. In conclusion, based on verifying the above hypotheses, this study aims to further explore the relationship between physical activity, rumination, anxiety, and depression of college students, analyze the relevant factors and mechanisms affecting depression of college students, and further reveal the chain mediating effect of rumination and anxiety between physical activity and depression among college students, to provide the theoretical basis for preventing and reducing depression in college students.

## Materials and methods

### Participants and procedure

The stratified cluster sampling method was used to conduct a questionnaire survey among college students at Zhaoqing University. A group test was conducted by class, and 6 classes were randomly selected from the four grades, that is, freshman, sophomore, junior, and senior. A total of 1,365 questionnaires were distributed through the Questionnaire Star applet. The questionnaires were distributed from 12 to 18 December 2022. After removing invalid questionnaires such as regular answers, missing data and reverse answers, a total of 1,292 data were collected, with an effective recovery rate of 94.7%. The age of the subjects ranged from 18 to 24 years old, and the average age was 19.40 ± 1.24 years old. There are 650 men and 642 women, including 344 freshmen, 325 sophomores, 309 juniors, and 314 seniors.

According to the Declaration of Helsinki, this study design has passed the ethical review of the Human Research Ethics Committee of Zhaoqing University (approval number: 2022-1205-01). Participants participated voluntarily, were informed of the purpose of the survey, answered methods and precautions, and signed informed consent forms. Before the test, the questionnaire data collectors are uniformly trained in the testing process, key points, and methods. In the test, mature scales are used for online measurement, questionnaires are sent to students through WeChat, and students complete the questionnaire independently on computers or mobile phones, emphasizing the principles of voluntary filling, the confidentiality of information, and anonymous filling.

### Physical activity questionnaire

The physical activity questionnaire of college students compiled by Chen et al. (2006) and revised by Wu (2016) was used to investigate the participation of college students in physical activity. The scale includes two dimensions, with 4 entries in each dimension. The scale uses a 5-point Likert score (1 = “strongly disagreed,” 5 = “strongly agreed”), taking the total score of the item. The higher the overall score, the higher the participant’s level of physical activity. Studies have shown that the scale has good reliability and validity among Chinese college students (Jiang et al., 2018). In this study, Cronbach’s α coefficient of the scale was 0.910. The fitting index of confirmatory analysis of the scale: χ^2^/df = 3.941, GFI = 0.986, AGFI = 0.973, IFI = 0.991, TLI = 0.987, CFI = 0.991, RMSEA = 0.049, indicating that the scale has good reliability and validity.

### Rumination scale

The Nolen-Hoeksema Rumination Scale (Nolen-Hoeksema and Morrow, 1991) revised by Han and Yang (2009) was used to measure college students’ rumination. The scale has 22 items, including symptom rumination, Brooding, and reflective reflection. A 4-pointLikert score (1 = never; 2 = sometimes; 3 = often; 4 = always) was adopted. The higher the score, the greater the tendency to ruminate. Studies have proved that this scale has good reliability and validity among Chinese college students (Wang et al., 2021). In this study, Cronbach’s α coefficient of the scale was 0.960. The fitting index of confirmatory analysis of the scale: χ^2^/df = 4.647, GFI = 0.909, AGFI = 0.889, IFI = 0.954, TLI = 0.949, CFI = 0.954, RMSEA = 0.062, indicating that the scale has good reliability and validity.

### Self-rating anxiety scale

The self-rating anxiety scale (SAS) developed by Zung (1971) was adopted. There are 20 items on the scale, using Likert 4 scale score, from 1 = no or little time to 4 = most or all time. Previous studies have shown that this scale has good reliability and validity and has been widely used (Shu et al., 2021). The higher the scale score, the more severe the anxiety symptoms. In this study, Cronbach’s α coefficient of the scale was 0.908. The fitting index of confirmatory analysis of the scale: χ^2^/df = 2.764, GFI = 0.994, AGFI = 0.983, IFI = 0.998, TLI = 0.997, CFI = 0.998, RMSEA = 0.043, indicating that the scale has good reliability and validity.

### Depression scale

In this study, the simplified depression scale compiled by Andresen et al. (1994) and revised by Xiong (2015) was adopted to measure the depression of college students. The scale includes three dimensions of physical symptoms, depressive mood, and positive mood, with a total of 10 measurement items. The scale uses a 4-point Likert score, ranging from “1 = none or almost none” to “4 = almost always.” The higher the score, the more severe the depression. The scale shows high reliability and validity among Chinese college students (Wang et al., 2022). In this study, Cronbach’s α coefficient of the scale was 0.917. The fitting index of confirmatory analysis of the scale: χ^2^/df = 3.950, GFI = 0.974, AGFI = 0.956, IFI = 0.987, TLI = 0.982, CFI = 0.987, RMSEA = 0.056, indicating that the scale has good reliability and validity.

### Statistical analysis

Confirmatory factor analysis was performed for all questionnaires using the software Amos26.0. Spss26.0 software was used to conduct descriptive statistics on the scores of each scale, and Pearson correlation analysis was used to test the correlation among physical exercise, rumination, anxiety, and depression. The Spss microprogram PROCESS Model 6 was used to examine the mediating role of rumination and anxiety between physical exercise and depression. The deviation-corrected Bootstrap method by repeated sampling 5,000 times was used to test the mediating effect of rumination and anxiety between physical activity and depression. Continuous variables are expressed as mean (M) ± standard deviation (SD). According to the references, *χ^2^/*df is less than 5, GFI, AGFI, IFI, TLI, and CFI are all greater than 0.8, and RMSEA is less than 0.08, which is acceptable. In this study, the significance level was set as *p* < 0.05.

## Results

### Common method bias test

Common method bias is the systematic error in index data results due to the same data acquisition method or the same measurement environment, which can generally be determined by the Harman single-factor test. When the variance explanation rate of the first factor in the results of the Harman single-factor test exceeds 40%, it can be considered that there is a large bias in the survey results of the commonly used methods (Podsakoff et al., 2003). The explanation rate of the first factor in this study is only 34.548%, lower than the test standard, so it can be considered that there is no serious common method bias effect in the results of this questionnaire.

### Descriptive statistics and correlation analysis

Table 1 shows the mean value, standard deviation, and correlation coefficients of physical activity, rumination, anxiety, and depression. The results suggest that all the variables are significantly corrected with each other. Physical activity is negatively correlated with rumination, anxiety, and depression, respectively (*r* = −0.322, *p* < 0.01; *r* = −0.313, *p* < 0.01; *r* = −0.399, *p* < 0.01). Both rumination and anxiety are positively correlated with depression (*r* = 0.434, *p* < 0.01; *r* = 0.429, *p* < 0.01). Rumination is positively correlated with anxiety (*r* = 0.354, *p* < 0.01).

**Table 1:** Means, standard deviations, and correlations among variables.

*Variable*	*M*	*SD*	*1*	*2*	*3*	*4*
1. Physical activity	28.93	7.385	1			
2. Rumination	43.10	13.172	−0.322^**^	1		
3. Anxiety	34.63	7.626	−0.313^**^	0.354^**^	1	
4. Depression	20.56	8.169	−0.399^**^	0.434^**^	0.429^**^	1

*N* = 1,292. ^**^p < 0.01; all tests were two-tailed.

### Examination of the mediating effects of rumination and anxiety

According to the suggestions of Wen and Ye (2014) on mediation effect testing, the chain mediation effect model is tested. With rumination and anxiety as the mediating variables, physical activity as the independent variable, and depression as the dependent variable, the stepwise regression method was used to test the mediating effect, and the test results are shown in Table 2.

**Table 2:** Regression analysis of variable relationships.

	Depression		Rumination		Anxiety		Depression	
	*β*	*t*	*β*	*t*	*β*	*t*	*β*	*t*
Physical activity	−0.399	−13.374***	−0.322	−10.440***	−0.222	−7.089^***^	−0.231	−7.940^***^
Rumination					0.283	9.017***	0.267	9.046^***^
Anxiety							0.262	8.902^***^
R	0.399		0.322		0.412		0.566	
R^2^	0.159		0.103		0.169		0.321	
F	178.873^***^		108.989^***^		96.284^***^		148.379^***^	

*** *p* < 0.001.

The results show that physical activity can significantly and negatively predict depression of college students (*β* = −0.399, *p* < 0.01). It indicates that the direct effect of physical activity on depression is significant. Therefore, hypothesis 1 is verified. After incorporating rumination into the regression equation, physical activity significantly and negatively predicts rumination (*β* = −0.322, *p* < 0.01), and rumination significantly and positively predicts depression (*β* = 0.267, *p* < 0.01). It can be concluded that rumination plays a partial mediating role between physical activity and depression. Hypothesis 2 is verified. After incorporating anxiety into the regression equation, physical activity also significantly and negatively predicts anxiety (*β* = −0.222, *p* < 0.01), and anxiety significantly and positively predicts depression (*β* = 0.262, *p* < 0.01). It can be concluded that anxiety plays a partial mediating role between physical activity and depression. Hypothesis 3 is verified. After integrating rumination and anxiety into the regression equation, rumination significantly and positively predicts anxiety (*β* = 0.283, *p* < 0.01), indicating the existence of chain mediation between rumination and anxiety. Hypothesis 4 is verified. It can be concluded that rumination and anxiety play a chain mediating role between physical activity and depression among college students.

**Figure 2:**
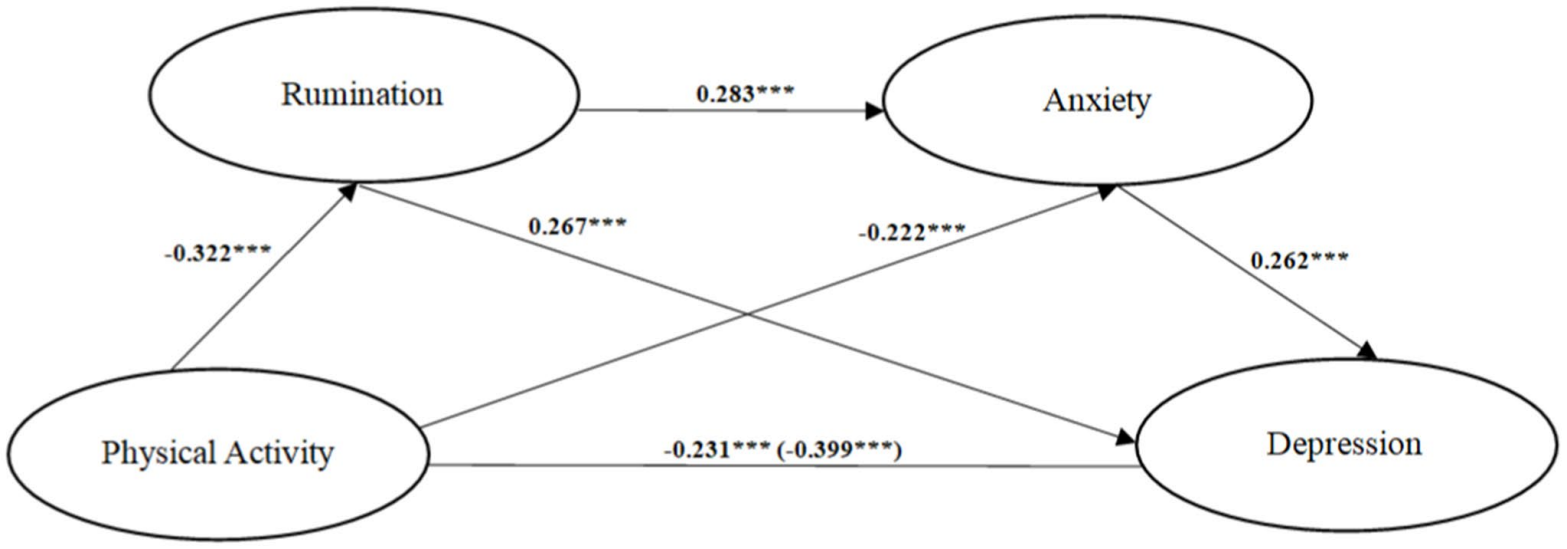
Chain mediation model of rumination and anxiety between physical activity and depression. ^***^p < 0.001.

Table 3 shows the mediating effect value of rumination and anxiety between physical activity and depression. The deviation-corrected percentile bootstrap method (repeated sampling 5,000 times) was used to test the indirect effects. The results show that the total standardized mediation effect of rumination and anxiety between physical activity and depression is −0.149. Specifically, the mediating effect included the following three pathways: physical activity → rumination → depression (−0.076), physical exercise → anxiety → depression (−0.052), physical activity → rumination → anxiety → depression (−0.021). The ratios of the three indirect effects to the total effect are 21.53, 14.59, and 5.98%, respectively. The 95% confidence interval of the three indirect effects did not contain the zero value, indicating that the three indirect effects were all significant. These results suggested that physical activity indirectly predicted depression not only through the independent mediating effect of rumination and anxiety but also through the chain mediating effect of rumination and anxiety. The independent mediating effect of rumination accounted for the highest ratio of the total indirect effect (21.53%). The mediating effect of rumination and anxiety on physical activity and depression is shown in Figure 2.

**Table 3:** Mediation effect analysis of rumination and anxiety.

		Effect	Boot SE	Boot LLCI	Boot ULCI	The ratio of indirect to total effect
Total effect		−0.353	0.030	−0.409	−0.292	
Direct effect		−0.204	0.033	−0.267	−0.138	57.90%
Indirect effect	Total indirect effect	−0.149	0.016	−0.181	−0.119	42.10%
	Indirect effect 1	−0.076	0.016	−0.109	−0.049	21.53%
	Indirect effect 2	−0.052	0.009	−0.070	−0.035	14.59%
	Indirect effect 3	−0.021	0.004	−0.030	−0.014	5.98%
	Compare 1	−0.025	0.021	−0.068	0.014	
	Compare 2	−0.055	0.015	−0.087	−0.029	
	Compare 3	−0.030	0.010	−0.050	−0.011	

Boot SE and Boot LLCI and Boot ULCI, respectively, refer to the standard error of effect and lower and upper limits of 95% confidence interval estimated by the Bootstrap deviation correction method. Indirect effect 1: Physical activity → rumination → depression; Indirect effect 2: Physical activity → anxiety → depression; Indirect effect 3: Physical activity → rumination → anxiety → depression.

## Discussion

### The relationship between physical activity and depression

This part examines a significant negative correlation between physical activity and depression of college students, consistent with the results of previous studies (Ghrouz et al., 2019; Zhang et al., 2022b). Hypothesis 1 is confirmed. Physical activity plays a crucial role in the prevention and treatment of depression (Paluska and Schwenk, 2000; Blake, 2012). Grasdalsmoen et al. (2020) found a negative correlation between depression and self-reported depression among university students. Ghrouz et al. (2019) pointed out that moderate and high-physical exercise levels were significantly negatively associated with scores for depression among university students. Luo et al. (2022) also found that physical activity effectively improved the symptoms of depression among university students. As suggested by Feng et al. (2014), high physical activity significantly reduced the prevalence of depressive problems among Chinese college freshmen. The more physical activities college students participated in, the lower their depression scores, and the higher their mental health levels (Liu, 2020). Physical activity that helps mitigate depressive-related symptoms optimizes brain-derived neurotrophic factor in key brain regions, and promotes neuronal health and recovery (Phillips, 2017). However, the physical activity plan adopted may affect its impact on depression. The exercise intensity influences the symptoms of depression among university students (Paolucci et al., 2018). Exercise helps ease symptoms of depression (Kvam et al., 2016), and larger effects were found for moderate intensity (Paolucci et al., 2018). The most common physical activity protocol adopted to improve major depressive disorder was the prescription of aerobic exercise at moderate intensity lasting 60 min per session, 3 times per week, for 24 weeks (De Sousa et al., 2021). Compared with controls, mind–body exercise showed the greatest improvement in symptoms of depression, followed by aerobic exercise and resistance exercise. Nevertheless, there was no statistically significant difference among the three types of exercise (Miller et al., 2020). Besides, greater frequency of physical activity is associated with lower odds of depression symptoms (D'Angelantonio et al., 2022). Patently, the effect of physical activity on college students’ depression still needs to be further studied.

### The mediating effect of rumination

This part analyzes the mediating role of rumination in the relation between physical activity and depression, thus Hypothesis 2 is validated, which is consistent with previous research findings that physical activity significantly and negatively predicts rumination (Schmitter et al., 2020) and rumination significantly and positively predicts depression (Lawrence et al., 2018). Rumination is one of the potential mediators and moderators explaining and specifying the impact of acute aerobic exercise on the subjective emotional recovery from subsequent stressors (Bernstein and Mcnally, 2017). This study examines three variables simultaneously, revealing that physical activity is an important predictor for improving rumination and reducing depression.

It is confirmed that physical activity can negatively predict rumination. Ye et al. (2022) claimed that physical activity significantly and negatively predicted the rumination of college students. Rumination significantly mediated the antidepressant effects of both Bikram yoga and aerobic exercise (La Rocque et al., 2021). A close relationship is found between physical exercise and rumination, where rumination served as an independent mediating variable in the association between physical exercise and mobile phone addiction (Zeng et al., 2022). A randomized controlled trial revealed that the mechanism by which physical activity therapy affects depression maybe mediated by rumination, self-esteem, and memory bias (Schmitter et al., 2020). People who participated in physical activities actively may be protected against the effects of rumination on hypothalamic–pituitary–adrenal axis reactivity, thus physical exercise moderates the influence of rumination on cortisol output trajectories (Puterman et al., 2011).

Moreover, rumination can positively predict depression, signifying that a decrease in rumination mitigates depression, consistent with previous research results (Liu et al., 2022). Rumination has been persistently implicated in the etiology of depression. According to the hopelessness theory of depression, some people have cognitive predispositions toward depression, and negative events can influence them through cognitive susceptibility (Abramson et al., 1989). Rumination is not only a negative cognitive pattern and coping style, but also a cognitive susceptibility factor leading to depression (Nolen-Hoeksema et al., 2008). Depression is characterized by difficulties in suppressing unrelated emotions, which are associated with rumination (Zetsche and Joormann, 2011). As one of the maladaptive emotion regulation strategies, rumination was found to be closely related to depression (Joormann and Gotlib, 2010; Tully et al., 2016). Rumination amplifies and prolongs negative emotions, ultimately triggering depression (Zhang and Zhou, 2018). As a negative personality trait, rumination is a catalyst for depression, signifying that rumination plays a moderating role in the process of depression induced by other risk factors. It is found that rumination has a significant predictive effect on depression (Lawrence et al., 2018). As stated by Wilkinson et al. (2013), high rumination predicts the onset of depressive disorder in healthy adolescents, while treatment of rumination may lead to a decline in the incidence and recurrence rates of depression. Many studies have explored the internal mechanisms of rumination on depression from the perspective of neurobiology (Tozzi et al., 2021). A meta-analysis of brain imaging studies confirmed the suspected association between rumination and the activation of default mode network, specifically implicating the default mode network core regions and the dorsal medial prefrontal cortex subsystem (Zhou et al., 2020). It is worth pointing out that rumination is a repetitive self-referential thinking style that is often interpreted as an expression of abnormalities of the default mode network observed during the “resting state” in major depressive disorder (Chen et al., 2020). Thus, lessening people’s tendency to engage in rumination may alleviate depressive symptoms. In this study, the independent mediating role of rumination is the most crucial indirect influencing indicator in the correlation between physical activity and depression. Therefore, it can be inferred that physical activity is more effective in alleviating depression by regulating college students’ rumination.

### The mediating effect of anxiety

It is found that anxiety plays a mediating role in the relationship between physical activity and depression, thus Hypothesis 3 is verified. Previous studies have also confirmed the findings that physical activity negatively predicts anxiety (Ghrouz et al., 2019; Chi et al., 2021), while anxiety positively predicts depression (Tiller, 2013; Bui and Fava, 2017). This paper investigates three variables simultaneously, revealing that physical exercise is an important predictor of reducing people’s anxiety, and an important factor in alleviating depression.

This part proves that physical activity negatively predicts the anxiety of college students. Studies have suggested that physical activity has anxiolytic effects. As stated by Chi et al. (2021), being moderately and highly physically active were associated with lower levels of anxiety symptoms. De Oliveira et al. (2019) investigated a negative correlation between low levels of physical activity and symptoms of anxiety and depression. Physical activity can effectively improve the situation of anxiety for college students (Luo et al., 2022). Ghrouz et al. (2019) revealed a significant negative correlation between physical activity and anxiety score among university students with moderate and high levels of exercise (Ghrouz et al., 2019). The more physical activities college students participated in, the lower their anxiety scores, and the higher their mental health levels (Liu, 2020). However, different modalities and predictors of change may influence the efficacy of physical activity in treating anxiety disorders. Several components of physical activity such as type, intensity, and time may influence the efficacy of physical activity interventions as treatment for anxiety disorders. It is well-established that aerobic exercise provides a positive benefit for treating anxiety (Archer et al., 2014), while some studies suggest that resistance exercise training significantly improves anxiety symptoms (Gordon et al., 2018, 2020). Both aerobic exercise and resistance training were efficacious in improving disorder status. Specifically, aerobic exercise improved general psychological distress and anxiety, while resistance training improved disorder-specific symptoms, anxiety sensitivity, and other aspects (LeBouthillier and Asmundson, 2017). Physical activity of different intensities significantly influenced the improvement of depression and anxiety among college students, however, physical exercise of 6 < 9 Mets intensity had a greater effect on anxiety than on depression (Luo et al., 2022). High-intensity exercise training showed greater effects than low-intensity training in treating anxiety (Aylett et al., 2018). Besides, there was a dose response to the anti-anxiety effect of physical activity of different intensities. The exercise at an intensity of 75% of the maximum heart rate had a better anti-anxiety effect compared with the exercise at an intensity of 45% and 60% of the maximum heart rate (Jin et al., 2002). However, different conclusions have been drawn in other studies. A systematic review and meta-analysis reported no beneficial effect of exercise on reducing anxiety and depressive syndromes (Pearsall et al., 2014). Thus, further research is needed on the impact of physical activity on depression.

This study confirms that anxiety is an important predictor of depression among college students. The interdependent relationship between depression and anxiety has been long been suggested and described. Clinical studies show that anxiety is accompanied by depression symptoms, and may be an expected precursor syndrome in the development of some forms of depression. Research findings show that depression and anxiety disorders are highly comorbid with, for example, 68% of patients with major depressive disorder also meet criteria for anxiety disorder. Empirical data suggest that depressive and anxiety disorders have common neurobiological pathways, including dysfunction in the serotonergic system that may explain the interdependent relationship between depression and anxiety (Bui and Fava, 2017). A survey revealed that 20.2% of Chinese college students had obvious symptoms of depression, 10.9% had obvious symptoms of anxiety, and 8.4% had both symptoms of depression and anxiety (Chen and Wu, 2021). According to a survey of Ethiopian university students, 35.4% experienced moderate-to-severe depressive symptoms and 48.4% had moderate-to-severe anxiety syndromes (Lemma et al., 2012). It can be concluded that some university students experience anxiety and depression at the same time, which greatly affect their physical and mental health. Thus, it is necessary to urge and encourage college students to actively engage in physical activity to prevent and improve their anxiety and depression levels.

### The chain mediation effect of rumination and anxiety

This study suggests a positive correlation between rumination and anxiety. Rumination has been proved to be an important causal variable of anxiety (Verstraeten et al., 2011; Hansell et al., 2022; Sun et al., 2022). As stated above, rumination is associated with anxiety in addition to depression. Since rumination involves the self-related aspects and self-focus is associated with anxiety, rumination is likely to have something to do with anxiety. The correlation between rumination and depression was 0.33, and between rumination and anxiety was 0.32. If the level of anxiety was controlled, the partial correlation between rumination and depression was 0.20; if the level of depression was controlled, the partial correlation between rumination and anxiety was 0.17 (Harrington and Blankenship, 2002). It can be seen that rumination is not only related to depression, but also has a positive effect on anxiety. Other studies also support a significant correlation between rumination and anxiety (Fresco et al., 2002). On this basis, this study constructs a chain mediation model to further explore the effect of physical activity on the depression among college students and its mechanism. According to the chain mediation model, rumination and anxiety play a mediating role between physical activity and depression among university students. Available evidence suggests that engaging in physical activity protects against rumination, anxiety, and depression. Hypothesis 4 proposed is confirmed. College students with a high level of rumination tend to dwell on their shortcomings, mistakes, the causes and consequences of negative events. Such a negative thinking mode aggravates their psychological and interpersonal pressure, and increases the level of anxiety. Also, they tend to develop depression and inferiority when communicating with others due to their low sense of self-identity, which greatly affects normal interpersonal communication and then induces psychological problems such as anxiety and depression. Physical activity helps cultivate an individual’s positive attitude, release negative emotions, reduce negative rumination, change negative beliefs, distract attention from negative thoughts and events and redirect attention to positive emotions, thereby alleviating anxiety and avoiding depression. Furthermore, the effects of physical activity on an individual’s self-concept (Zhang et al., 2022a), social support (Zhang et al., 2022a), sleep quality (Hao et al., 2023), physical satisfaction (Hao et al., 2023), mood (Miller et al., 2019), and self-efficacy (Miller et al., 2019) is beneficial for reducing depression syndromes. Physical activity can improve the depression among college students by reducing rumination and anxiety. To sum up, rumination and anxiety play a chain mediating role between physical activity and depression.

### Practical significance

This study explores the relationship between physical activity and depression of college students, as well as the mediating effect of rumination and anxiety between them. The results show that physical activity not only directly and negatively predicts anxiety but also indirectly and negatively predicts depression through the independent mediating effect of rumination and anxiety, as well as the chain mediating effect of rumination and anxiety. This paper reveals the influence of physical activity on the depression of college students and its possible mechanism, which has a certain guiding significance for the prevention and intervention of depression in college students. The results of this study suggest that we should not only pay attention to the direct influence of physical activity on the depression of college students but also improve anxiety by reducing rumination of college students through physical activity, to alleviate depression of college students. In practical application, depression of college students can be improved by the following measures: First, encourage college students to participate in physical activity, provide relevant scientific physical activity guidance, improve the rumination, anxiety, and depression of college students, and then improve the mental health level of college students. Secondly, rumination and anxiety are important mediating factors affecting college students’ depression. We should attach importance to the rumination and anxiety of college students. Methods such as mindfulness-based decompression, mindfulness meditation, and positive rumination training can be used to help college students cultivate a positive attitude, reduce the influence of negative emotions and produce positive rumination thinking, relieve anxiety, and prevent and alleviate depression.

### Limitations and prospects

The findings of this study have certain theoretical value and practical guidance but also have some limitations. First, this study adopts the self-report method for the questionnaire survey, and there may be some deviation between the self-reported information and the actual information. Future research may try to collect data from multiple approaches. Second, the cross-sectional study was adopted in this study to explore the influence of physical activity on the depression of college students and its possible mechanism. However, this research method could not infer the causal relationship between variables, and longitudinal research or experimental research could be used to further verify the conclusions of this study. In addition, this study only considered the mediating effect of rumination and anxiety on physical activity and depression, but in fact, there may be other mediating variables such as perceived social support, self-esteem, peer relationship, etc., which need to be further explored.

## Conclusion

(1) Physical activity can significantly and negatively predict the rumination, anxiety, and depression of college students, and physical activity may help improve the rumination, anxiety, and depression of college students. (2) Physical activity can not only directly predict depression of college students, but also indirectly predict depression of college students through the independent mediating effect of rumination and anxiety, and indirectly predict depression through the chain mediating effect of rumination and anxiety. It is suggested that in improving and intervening in the depression of college students, we should not only pay attention to enhancing physical activity but also pay special attention to reducing the rumination and anxiety of college students.

## Data availability statement

The original contributions presented in the study are included in the article/Supplementary material, further inquiries can be directed to the corresponding author.

## Author contributions

YL designed the study, analyzed the data, and wrote the manuscript. KG revised the manuscript. QF collected the data. All authors contributed to the article and approved the submitted version.

## Funding

This research was funded by the Construction and practice of a practical teaching system of Physical Education Specialty in local Normal Colleges under the background of “New Normal” construction (2021GXJK482); the Construction and practice of a practical teaching system of physical education Specialty in local Normal Colleges under the Background of University Transformation (Guangdong Higher Education Letter [2023] No.4-962); and the Construction and practice of practical teaching system of physical education specialists in local Normal colleges under the background of university transformation (zlgc202135).

## Conflict of interest

The authors declare that the research was conducted in the absence of any commercial or financial relationships that could be construed as a potential conflict of interest.

## Publisher’s note

All claims expressed in this article are solely those of the authors and do not necessarily represent those of their affiliated organizations, or those of the publisher, the editors and the reviewers. Any product that may be evaluated in this article, or claim that may be made by its manufacturer, is not guaranteed or endorsed by the publisher.
